# Changes in soil ecological stoichiometry and microbial communities related to leaf stoichiometry of different halophytes

**DOI:** 10.3389/fpls.2026.1793742

**Published:** 2026-05-08

**Authors:** Yaqing Pan, Zixuan Chen, Juan Chen, Qing Wang, Xigang Liu, Zongxiao Zhang, Ayala Bahetijiang, Jinpeng Hu, Peng Kang

**Affiliations:** 1 Xinjiang Laboratory of Lake Environment and Resources in Arid Zone, College of Geographic Science and Tourism, Xinjiang Normal University, Urumqi, China; 2 Key Laboratory of Soil Ecological Health and Microbial Regulation, Ningxia Normal University, Guyuan, China; 3 College of Pastoral Agriculture Science and Technology, Lanzhou University, Lanzhou, China

**Keywords:** halophytes, leaf stoichiometry, salt island, soil ecological stoichiometry, soil microbial community, co-occurrence network

## Abstract

Species differences among halophytes in arid regions, along with alterations in the physicochemical properties of understory soils induced by their litterfall, often shape distinct salt island effects. In this study, we investigated the soil physicochemical properties and leaf traits of *Kalidium foliatum (KFSI)*, *Nitraria tangutorum* (NTSI), *Reaumuria songarica* (RSSI), and *Tamarix hohenackeri* (THSI) in arid salt marshes, and profiled soil bacterial and fungal communities using high-throughput sequencing. Along the salt island successional sequence from KFSI to NTSI to RSSI to THSI, soil water content, pH, electrical conductivity, and Na⁺ and K⁺ contents decreased successively. The leaf carbon content of the four halophytes was found to be negatively correlated with soil salt ions, whereas nitrogen and phosphorus contents were positively correlated with salinity indicators. THSI had the highest total carbon and soil organic carbon content, which were 117% and 280% higher than those of KFSI, respectively. The Shannon index of soil bacteria in THSI was higher, and the complexity of its microbial network (1,305 nodes, 3,929 edges) was significantly greater than that of the other salt islands. In addition, random forest analysis showed that soil physical and chemical properties (accounting for 26 -58% of the total) and plant characteristics (15 -26%) jointly regulate soil ecological stoichiometric ratios. Soil physical properties positively drove the complexity of the microbial network by influencing the chemical composition of plant leaves and microbial community composition (p < 0.01). This study delineates successional gradients in soil properties, plant leaf traits, and microbial communities across halophyte_specific salt islands and clarifies how their interplay regulates soil ecological stoichiometry, thereby shaping ecosystem structure and function in arid saline environments.

## 1 Introduction

Desert plants, particularly shrubs that dominate desert plant communities, have evolved unique fitness mechanisms in saline and arid environments (Knapp et al., 2008). Owing to their well-developed root systems, they can draw sufficient water and nutrients for survival (Eldridge et al., 2011). Under prolonged wind and sand impact and through the action of their root systems, desert shrubs immobilize mobile sandy soils, forming small, stable islands in deserts (Meglioli et al., 2017). The input of litter on the ground and root exudates of desert shrubs increases the accumulation of nutrients in the plant canopy, resulting in so-called “fertilizer islands” (Ridolfi et al., 2008). In desert ecosystems, seasonal precipitation accumulates in the lower terrain, forming salt marshes of different sizes. Similarly, halophytes appear as the dominant plants in these salt marsh wetlands (Pan et al., 2021; Zhang et al., 2022). Evaporation of water from salt marsh wetlands causes salt from the soil to accumulate at the surface. Salt-dilution halophytes store root-absorbed Na^+^ in cellular vacuoles in succulent leaves; thus, when leaves are subjected to external forces or aging, the salts accumulated in the leaves enter the soil (Flowers and Colmer, 2008; Zhao et al., 2011; Pan et al., 2024a). Salt-secretion halophytes excrete Na^+^ absorbed in the roots through leaf salt glands/salt bladders, and the excreted salts continue to circulate within the rhizosphere-plant-leaf system (Yuan et al., 2016; Dassanayake and Larkin, 2017; Qu et al., 2024). Whether halophytes are of the salt-dilution or salt-secretion type, the salt absorbed by the plant root system is eventually added to the surface of the fertilizer island through the medium of leaves, thus producing the “salt island effect”. While both phenomena highlight plants as ecosystem engineers, the specific mechanisms underlying salt island formation, particularly the role of plant functional traits and their interplay with soil biota, remain less elucidated than those for fertile islands.

Fertilizer islands are known to result from the shrub-driven enrichment of soil nutrients in deserts (Breshears et al., 2009). Fertilizer islands often contain high amounts of organic matter and nutrients (Li et al., 2021). In addition, fertilizer islands have higher microbial biomass and activity than the surrounding area, thus enhancing soil nutrient metabolism and cycling (Gao et al., 2022; Liu et al., 2024). The salt island effect is described in a similar way as the fertilizer island effect: in addition to soil nutrient enrichment by the plants, the halophytes associated with salt islands have an effect on soil water content (SWC), electrical conductivity (EC), concentration of salt ions (including Na^+^, K^+^, Ca^2+^, Mg^2+^, and SO_4_^2−^), and other factors (Yang B. et al., 2018). Studies on halophyte-soil ecological functions have often focused on how changes in plant communities affect soil nutrient changes, emphasizing the comprehensive relationship between halophyte cover and regional ecosystems (Pan et al., 2021, 2024b). Critically, these plant-derived inputs do not act in isolation; they are processed and modulated by soil microbial communities. Microbial biomass, activity, and community composition are pivotal in regulating decomposition, nutrient mineralization, and the stabilization of soil organic matter, ultimately determining the net outcome of plant inputs on island soil properties (Delgado-Baquerizo et al., 2017; Liu et al., 2024).

The physical and chemical properties of shrub soils in desert ecosystems are largely driven by plant characteristics (eg. leaf characteristics) which alter the intensity of plant inputs of carbon, nitrogen, and other nutrients to the soil, thereby disrupting the balance of plant-soil nutrient cycling (Delgado-Baquerizo et al., 2017). Additional inputs of carbon and nitrogen from plant leaves promote the formation of specialized microbial communities, and this increase in biodiversity mediates plant–soil nutrient coupling (Elser et al., 2011; He et al., 2011). Notably, the functioning of soil microbial communities extends beyond biomass and activity; their compositional structure and inter-species interactions are crucial (Mooshammer et al., 2012). Microbial co-occurrence networks reflect the complexity and stability of ecological interactions, and their complexity has been strongly linked to ecosystem processes and soil ecological stoichiometry (Hao et al., 2023; Kang et al., 2025). In the context of fertile or salt islands, different halophyte species, through distinct leaf litter chemistry and rhizodeposits, are likely to shape unique soil nutritional and saline niches. These niches, in turn, should select for specific microbial assemblages and shape their interaction networks. Yet, how the microbial co-occurrence network within salt islands responds to variations in halophyte leaf stoichiometry and associated soil properties is virtually unknown. This represents a critical gap, as understanding these microbe-mediated linkages is essential to predict the resilience and biogeochemical functioning of salt marsh ecosystems under changing environments.

Plant growth and development depend on soil nutrient supply, forming a tightly coupled system. Changes in the direction and intensity of plant inputs to soil energy (litters and root exudates) directly affect the physical and chemical properties of soil under the canopy. In previous studies on salt islands, the interrelationships between leaf functional traits and salt islands have often been neglected (Pan et al., 2021). Therefore, to bridge these knowledge gaps, we conducted an integrated study in the Beichi salt marsh of Ningxia, a site featuring distinct halophyte species (*Kalidium foliatum*, *Nitraria tangutorum*, *Reaumuria songarica*, and *Tamarix hohenackeri*), each forming a discernible salt island. By simultaneously analyzing plant leaf ecological stoichiometry, soil physico-chemical properties, and employing high-throughput sequencing of soil microbial communities, we aimed to unravel the interrelationships among halophyte traits, salt island soils, and microbial networks. Specifically, we tested the following hypotheses: (1) Significant interspecific differences in leaf ecological stoichiometry (C:N:P ratios) among halophyte species, reflecting distinct nutrient use strategies, lead to corresponding variations in the soil nutrient content (e.g., SOC, TN, TP) and stoichiometry (C:N:P) of their associated salt islands. (2) The structure and complexity of soil microbial co-occurrence networks differ significantly among salt islands formed by different halophyte species, and these variations in microbial network complexity are significantly correlated with the ecological stoichiometry of both halophyte leaves and salt island soils.

## 2 Materials and methods

### 2.1 Study area and sample collection

The study site is located in the Beichi salt marsh (E106°38′-106°40′; N37°28′-37°29′), northwest of Huianpu Town, Ningxia, China, and covers an area of approximately 27 km^2^. The Beichi salt marsh is part of an inland saline lake, where the salt-dilution halophytes *K. foliatum*, and *N. tangutorum*; and the salt-secretion halophytes *R. songarica* and *T. hohenackeri* are distributed. The region is a desert steppe ecosystem with an average temperature of 11.3 °C and an average annual precipitation of 21.35 cm (as recorded in 2024).

Within the study area, four sampling zones were delineated from the direction of sampling is from upland to lowland, with each sampling zone located more than 100 m away from the others. The transect begins in the high marsh (near dry land) and progresses to the low marsh and open water. Within each area, the subcanopy soil was collected from *K. foliatum* (KFSI), *N. tangutorum* (NTSI), *R. songarica* (RSSI), and *T. hohenackeri* (THSI) (**Supplementary Figure S1**). Soil samples were collected from a depth of 0–20 cm in the east, west, north, and south directions beneath the canopies of four halophytes. We combined the soil samples collected from beneath each plant’s canopy into a single sample, with nine replicates per plant species, for a total of 36 samples. Additionally, we collected the leaves of each of the four halophytes when collecting the soil samples.

### 2.2 Analysis of physical and chemical properties of plants and soils

The SWC of the soil samples was determined by weighing 100 g of fresh soil before and after air-drying. Soil pH and EC were determined using air-dried soil at a soil-water ratio of 1:5. In this experiment, 20 g of air-dried soil was collected and 100 mL of deionized water was added and stirred thoroughly, after which it was analyzed using a pH meter (LeiCi PHS-3E, Shanghai, China) and an EC meter (Leici DDS-307A, Shanghai, China) (Bao, 2000). Total soil carbon (TC), total nitrogen (TN), and leaf carbon and nitrogen contents were determined using an elemental analyzer (Elementar Vario MACRO Cube, Germany). The soil total phosphorus (TP) and leaf phosphorus content was determined by vanadium-molybdenum-yellow absorbance spectrophotometry after decoction with concentrated sulfuric acid (Bao, 2000). The soil organic carbon (SOC) content was determined using an automatic organic carbon analyzer (Shimadzu TOC-VCPH, Japan). Air-dried soil was extracted by adding K_2_SO_4_ (Bolan et al., 1996), and dissolved organic carbon (DOC; Shimadzu TOC-VCPH, Japan) and dissolved organic nitrogen (DON; Thermo Fisher FlashSmart, Germany) were determined. The soil available phosphorus (OP) content was determined after air-drying the soil and shock-extraction by adding NaHCO_3_ and molybdenum-antimony reagents (Olsen, 1954). The Na^+^ and K^+^ contents of the air-dried soil were measured using flame photometry, Ca^2+^ and Mg^2+^ contents were determined using atomic absorption spectrophotometry, and soil SO_4_²^-^, HCO_3_^-^, and Cl^-^ contents were determined using titration (Bettinelli et al., 2000), the leaf Na^+^ and K^+^ contents measured in the same manner. Statistical analysis of the data in this study was performed using ANOVA.

### 2.3 Soil DNA extraction and sequencing

Each of the 36 fresh soil samples (1.0 g) were added: 100 mmol/L Tris-HCl, 100 mmol/L EDTA, 200 mmol/L NaCl, 1.0% PVP, and 2.0% CTAB, pH 8.0. The mixture was boiled, cooled, and centrifuged. After extracting the supernatant, a solution (100 mM Tris-HCl, 200 mM NaCl, and 2.0% SDS, pH 8.0) was added, and the supernatant was collected after centrifugation for gel purification. The 16S rRNA gene V3–4 regions of 36 eligible samples were amplified using the primers 341F (5′-CCTAYGGGRBGCASCAG-3′) and 806R (5′-GGACTACNNGGGTATCATAT-3′), respectively (Claesson et al., 2009). In addition, the ITS1–2 gene regions of 36 eligible samples were amplified using primers ITS1 (5′-CTTG GTCA TTTA GAGG AAGT AA-3′) and ITS2 (5′-GCTG CGTT CTTC ATCG ATGC-3′) (De Beeck et al., 2014).

After PCR amplification, the raw data were sequenced using Illumina NovaSeq 6000 platform and spliced using FLASH (V1.2.11) software (Magoč and Salzberg, 2011). Valid data were obtained by filtering the chimeras using Usearch software (Haas et al., 2011). Operable classification units (OTUs) were obtained by noise reduction of valid data using the DADA2 module in QIIME (Version 1.9) software (Callahan et al., 2016). Lastly, the representative sequence of each OTU was annotated by species using the classification sklearn algorithm in QIIME (Bokulich et al., 2018). Finally, the data of each sample were normalized. The normalization was performed based on the sample with the smallest data volume as the standard, while the subsequent alpha and beta diversity analysis were all conducted based on the data after normalization. We further annotated the OTUs using species annotation databases selected from SILVA 138 (bacteria) and UNITE 8.3 (fungi) (Koljalg et al., 2005; Yilmaz et al., 2014). Raw bacterial and fungal data of soil samples from this study were submitted to the NCBI database (PRJNA1210856 and PRJNA1211016).

### 2.4 Data calculation

Various parameters of the plant leaves and the physical and chemical properties of the soil were analyzed. Then, leaf stoichiometry, soil nutrient resource stoichiometry, and soil matrix stoichiometry were calculated as described previously (Hao et al., 2023). Leaf Stoichiometryr reflects the elemental ratios (C:N, C:P, N:P) within the plant tissue itself, calculated using the TC,TN, and TP concentrations of the leaves.

Leaf C:N (or C:P, N:P) = Leaf TC/TN (or TC/TP, TN/TP) (1)

Soil Nutrient Resource Stoichiometry reflects the elemental ratios of the labile, bioavailable nutrient pool readily accessible for microbial uptake and short-term nutrient cycling. It was calculated using DOC, DON, and OP.

Resources C:N (or C:P, N:P) = DOC/DON (or DOC/OP, DON/OP) (2)

Soil Matrix Stoichiometry reflects the elemental ratios of the total soil nutrient capital. It was calculated using SOC, TN, and TP.

Soil C:N (or C:P, N:P) = SOC/TN (or SOC/TP, TN/TP) (3)

### 2.5 Data analysis

The relationships between Leaf C, N, and P and soil physicochemical parameters were visualized using “ggplot2” package in R software (4.1) (Racine, 2012). Spearman’s correlation coefficients between plant leaf stoichiometry and soil environmental variables were displayed in a matrix heat map using “linkET” package (Sunagawa et al., 2015). Alpha diversity indices, including Shannon and abundance-based coverage estimator (ACE) indices, were calculated for bacterial and fungal communities in the 36 soil samples using QIIME (v1.9.1) (Caporaso et al., 2011; Caporaso et al., 2012). Based on the OTU relative abundance table, the Bray-Curtis distance matrix was calculated to evaluate the differences in microbial community composition among the samples. Visualization was performed using nonmetric multidimensional scaling (NMDS) analysis. To test whether the differences in community structure among different halophyte were statistically significant, we conducted multivariate permutation variance analysis. The relationship between soil bacterial and fungal diversity indices and soil environment was characterized using “ggplot2” package. “galluvial” package was used to demonstrate the composition of the dominant phyla (TOP 10) of soil bacteria and fungi from the different halophytes. The importance of dominant phyla for environmental variables was also assessed using “randomForest” package, the model was run 1000 times to obtain stable importance estimates (Jiao et al., 2018).

Co-occurrence networks were used to demonstrate the interrelationships between bacterial and fungal OTUs in the salt island soils of different halophytes. We separately calculated the spearman correlation coefficient (|r| < 0.9, *p* < 0.01) for bacterial-fungal OTUs, and visualized the data using Cytoscape software (version 3.7.1) (Shannon et al., 2003). In addition, we calculated the subnetwork parameters (nodes, edges, average density, transitivity, diameter, and average path length) for bacteria and fungi separately from 36 soil samples (Ma et al., 2016); the correlations with the major phyla were assessed through redundancy analysis (RDA). Correlation heat maps were also drawn for the subnetwork parameters and soil environment variables. Lastly, we calculated the network complexity index (NCI) and constructed partial least squares path models (PLS-PM) using “plspm” package to predict the relationship between soil physical properties, soil matrix stoichiometry, soil resource stoichiometry, and microbial communities (Hair et al., 2012). In this study, all parameters were described by one-way analysis with Duncan’s multiple range test for significant differences between groups (*p* < 0.05).

## 3 Results

### 3.1 Changes in physical and chemical characteristics of salt islands under different halophytes

With the outward extension of the salt marsh, soil SWC, pH, and EC exhibited decreasing trends from KFSI to THSI. The soil concentrations of Na^+^, K^+^, Cl^-^, and SO_4_²^-^ decreased accordingly. Simultaneously, KFSI near the salt marsh showed higher TN and DON contents, exceeding those of distant THSI by 49.90% and 28.55%, respectively. Conversely, THSI had higher TC, SOC, TP, and AP contents, surpassing those of KFSI by 1.18 times, 2.80 times, 58.19%, and 88.74%, respectively (**Table 1**).

**Table 1:** Soil physicochemical characteristics of different halophytes in salt islands.

Soil physicochemical properties	KFSI	NTSI	RSSI	THSI
pH	9.09 ± 0.20a	8.29 ± 0.10b	7.72 ± 0.06c	7.33 ± 0.11d
SWC (%)	30.80 ± 1.91a	19.57 ± 1.25b	15.54 ± 1.21c	12.01 ± 0.81c
EC (μs/m)	4598.55 ± 452.80a	1387.42 ± 108.09b	539.25 ± 27.80c	386.29 ± 21.65c
Na^+^ (g/kg)	6.97 ± 0.59a	4.06 ± 0.47b	2.47 ± 0.29c	1.24 ± 0.18d
K^+^ (g/kg)	0.06 ± 0.002a	0.06 ± 0.003a	0.04 ± 0.006b	0.04 ± 0.008b
Ca^2+^ (g/kg)	1.60 ± 0.37a	2.34 ± 0.13a	1.96 ± 0.21a	1.82 ± 0.35a
Mg^2+^ (g/kg)	1.04 ± 0.15a	1.29 ± 0.20a	0.20 ± 0.02b	0.19 ± 0.04b
Cl^-^ (g/kg)	9.10 ± 0.49a	5.81 ± 0.50b	2.19 ± 0.23c	1.05 ± 0.07d
SO2- 4 (g/kg)	8.90 ± 0.97a	7.75 ± 0.82a	4.92 ± 0.36b	2.66 ± 0.48c
TC (g/kg)	3.84 ± 0.49c	6.59 ± 0.44b	7.10 ± 0.67ab	8.35 ± 0.44a
SOC (g/kg)	1.02 ± 0.14c	2.15 ± 0.29b	2.27 ± 0.26b	3.88 ± 0.30a
TN (g/kg)	0.68 ± 0.04a	0.49 ± 0.03b	0.46 ± 0.04b	0.45 ± 0.02b
TP (g/kg)	0.24 ± 0.02b	0.23 ± 0.03b	0.31 ± 0.05ab	0.37 ± 0.01a
DOC (mg/kg)	74.19 ± 3.97b	100.87 ± 5.16a	89.69 ± 3.54a	98.85 ± 6.60a
DON (mg/kg)	1.56 ± 0.12a	1.27 ± 0.14ab	1.13 ± 0.07b	1.22 ± 0.12b
AP (mg/kg)	0.90 ± 0.09b	1.56 ± 0.17a	1.59 ± 0.21a	1.70 ± 0.10a

The data were presented as mean ± standard error (n = 9). Different letters in each column indicate significant differences at the *p* < 0.05 level (Duncan test). The following table was the same.

### 3.2 Relationship between leaf chemical properties of halophytes and environmental variables

**Figure 1:**
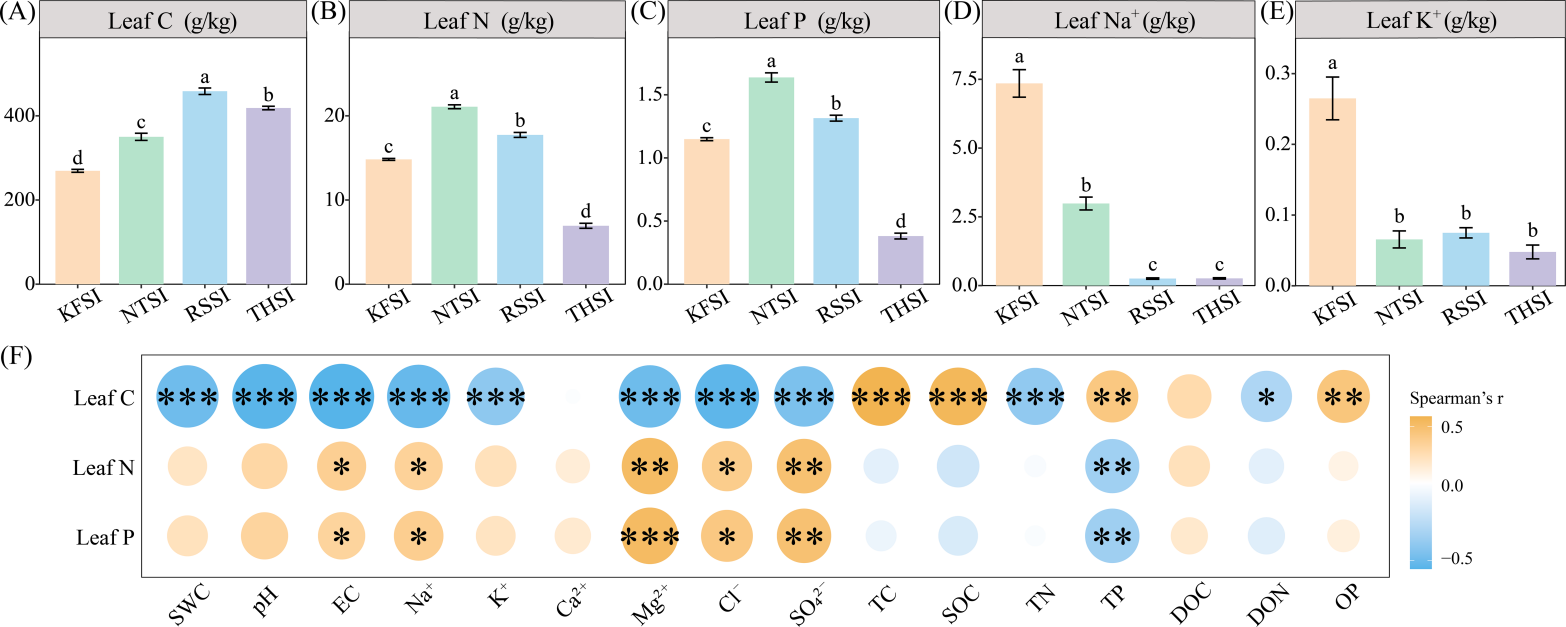
Leaf carbon **(A)**, nitrogen **(B)**, phosphorus **(C)**, Na^+^ **(D)**, and K^+^ **(E)** contents of four halophytes, and the relationship with soil physicochemical properties of salt island **(F)**. Different lowercase letters indicate significant differences (*p < 0.05, **p < 0.01, and ***p < 0.001).

**Figure 2:**
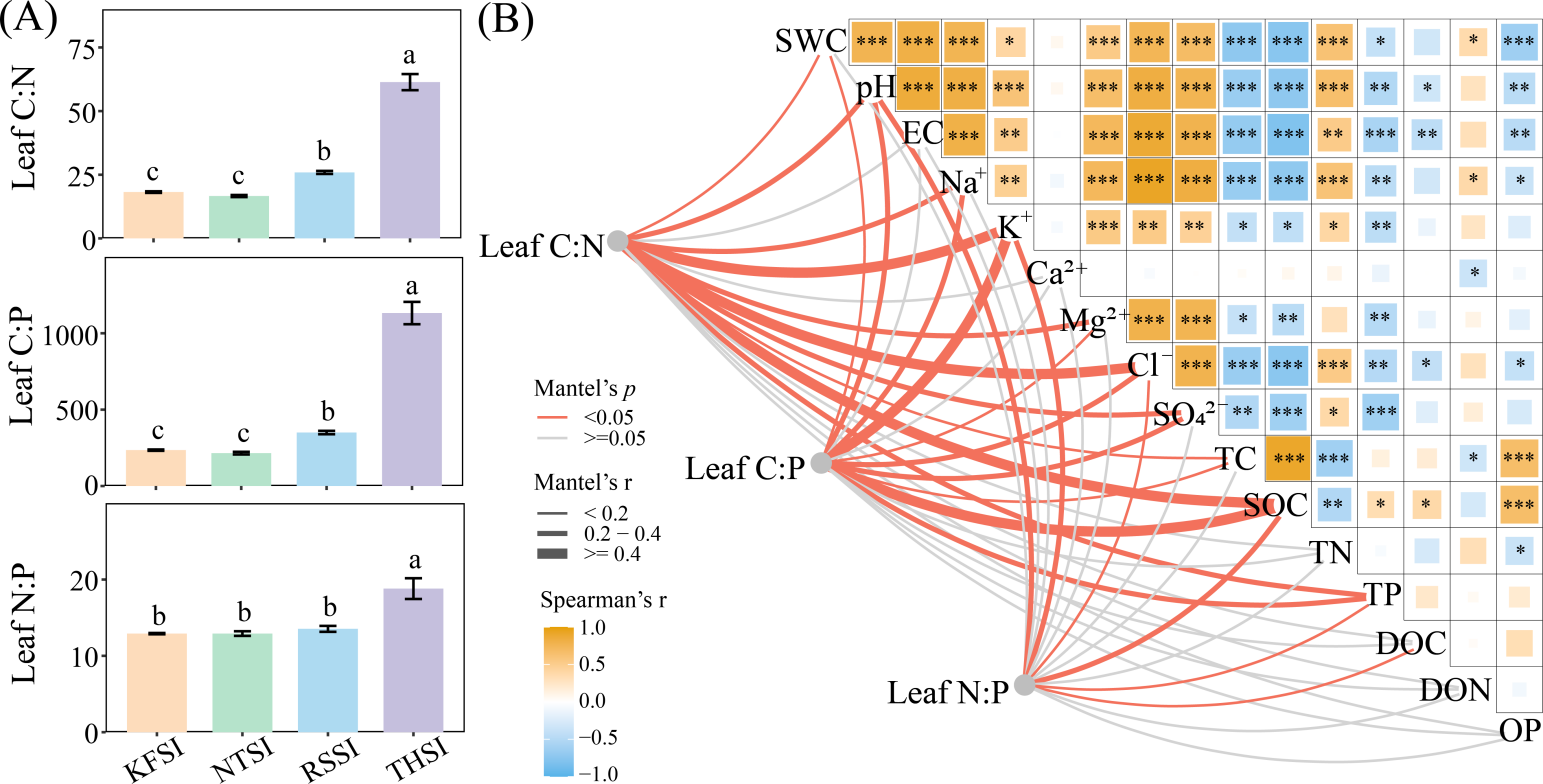
Leaf C:N:P stoichiometric characteristics of four halophytes **(A)** and their relationship with soil physicochemical properties of each salt island **(B)** (*p < 0.05, **p < 0.01, and ***p < 0.001).

Differences in halophyte types can lead to changes in leaf carbon, nitrogen, and phosphorus contents. The leaves of *R. songarica* had a higher carbon content (458.41 g/kg), whereas the leaves of *N. tangutorum* had higher nitrogen (21.07 g/kg) and phosphorus content (1.64 g/kg). The leaves of *K. foliatum* had higher concentrations of Na^+^ (7.35 g/kg) and K^+^ (0.26 g/kg) than what was found in samples from the other halophytes. Physicochemical analysis of salt island soil revealed that the leaf carbon content of halophytes was significantly and negatively correlated with SWC, pH, EC, Na^+^, K^+^, and TN. In contrast, leaf nitrogen and phosphorus were positively correlated with saline island EC, Na^+^, Mg^2+^, Cl^-^, and SO_4_^2-^ (**Figure 1**). Additionally, the Leaf C:N, C:P, and N:P ratios of *T. hohenackeri* were higher than in the other plants. The Mantel test analysis revealed that the Leaf C:N, C:P, and N:P ratios of all halophytes were correlated with pH, K^+^, Cl^-^, SOC, and TP content in the salt island (**Figure 2**).

### 3.3 Factors influencing soil C:N:P ecological stoichiometry

**Figure 3:**
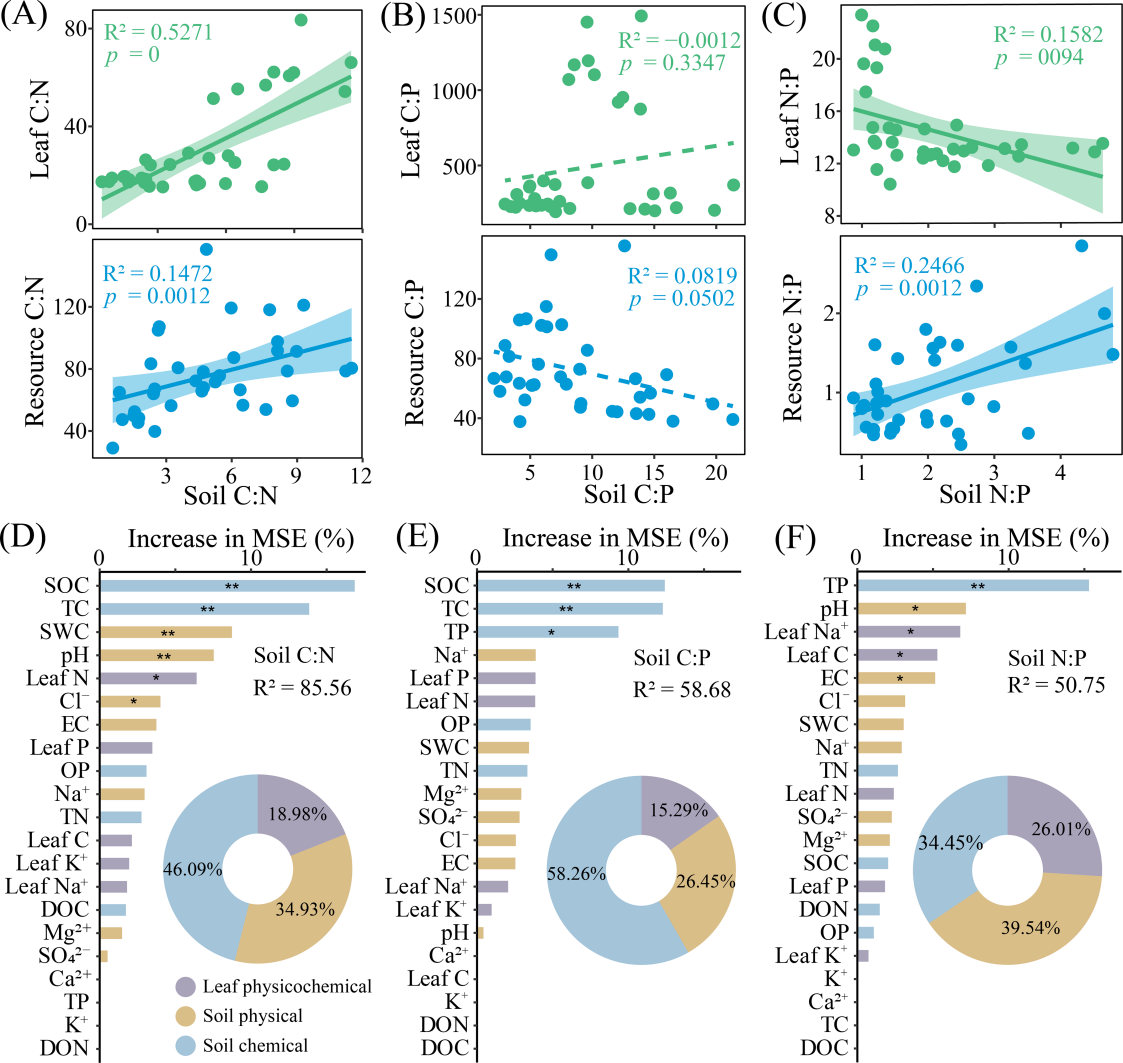
The linear relationships between the C:N **(A)**, C:P **(B)**, and N:P **(C)** ratio of soil and plant leaves as well as resources in different halophytes. Random forest interpretation of soil physicochemical properties and plant leaf chemical characteristics on soil C:N **(D)**, C:P **(E)**, and N:P (**F**) ratios.

In terms of the ecological stoichiometry of the salt islands, THSI had a higher Soil C:N ratio, whereas KFSI had higher ratio of Soil N:P, Resources C:P and N:P (**Supplementary Table 1**). The Leaf C:N and Resources C:N ratios of halophytes had a linear relationship with the Soil C:N ratio; similarly, the Leaf N:P and Resources N:P ratios were also linearly related to the Soil N:P ratio (**Figures 3A-C**). Concurrently, the random forest analysis revealed that soil physical properties (34.93%, 26.45%, and 39.54), soil chemical properties (46.09%, 58.26%, and 34.45%), and halophyte leaf characteristics (18.98%, 15.29%, and 26.01%) were the key variables influencing soil ecological stoichiometry (Soil C:N, C:P, and N:P) in the salt islands (**Figures 3D-F**).

### 3.4 Differences in soil bacterial and fungal communities and the influencing factors

The soil bacterial Shannon index from the THSI samples was higher than that of the other three salt islands, and it also exhibited higher fungal Shannon and ACE indices. Based on NMDS analysis, significant differences were observed between the bacterial and fungal communities across the salt islands (stress < 0.2, *p* = 0.001) (**Supplementary Figure S2**). Furthermore, we examined the correlations between bacterial and fungal community diversity (Shannon, ACE, NMDS1, and NMDS2 indices) and halophyte leaf nitrogen, phosphorus, Na^+^, and K^+^; soil SWC, EC, Na^+^, and Cl ^-^; and soil SOC, Soil C:N, Soil C:P, Resources C:N, and Resources N:P (**Supplementary Figure S3**).

**Figure 4:**
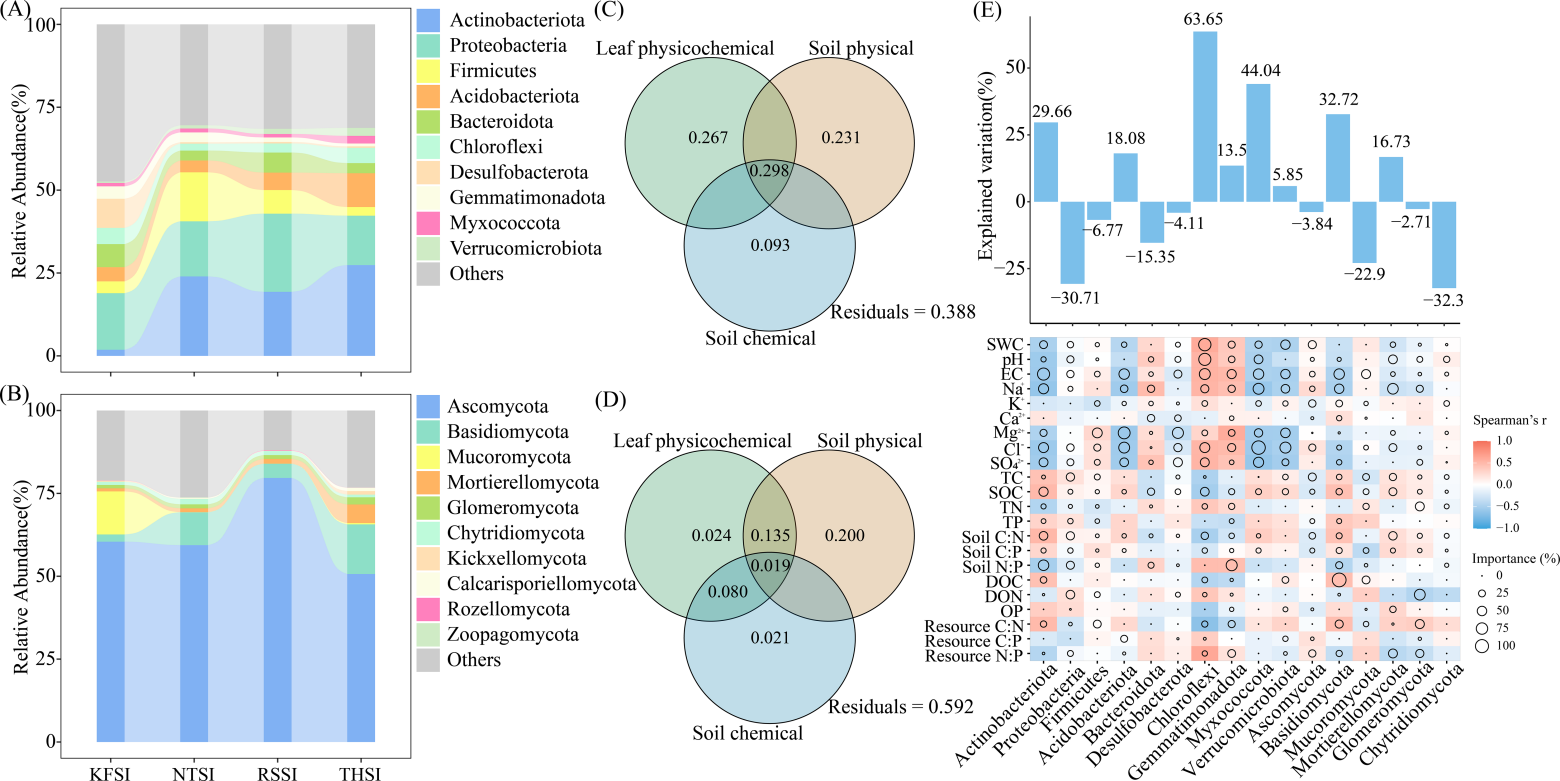
Relative abundance of the top 10 bacterial **(A)** and fungal **(B)** phyla in salt island soils. Variation in the plant leaf chemical characteristics and soil physicochemical characteristics in relation to the top 10 bacterial **(C)** and fungal **(D)** phyla is also shown, along with ecological chemical metrics in the random forest analysis **(E)**.

The soil bacteria on salt islands are primarily composed of the phyla Actinobacteriota, Proteobacteria, Firmicutes, Acidobacteriota, Bacteroidota, and Chloroflexi, whereas the fungi consist of Ascomycota, Basidiomycota, and Mucoromycota. The chemical characteristics of halophyte leaves (0.267 and 0.024), soil physical properties (0.231 and 0.200), and chemical properties (0.093 and 0.021) explained the changes in bacterial and fungal community compositions. Random forest analysis indicated that Chloroflexi (63.65%), Myxococcota (44.04%), Basidiomycota (32.73%), Actinobacteriota (29.66%), Acidobacteriota (18.08%), and Mortierellomycota (16.73%) exhibited higher explanatory rates for changes in the soil physicochemical and ecological stoichiometry (**Figure 4**). Further analysis revealed that the relative abundances of Acidobacteriota, Myxococcota, Basidiomycota, and Mortierellomycota in the soil of THSI were significantly higher than those of the other halophytes on the salt islands (**Supplementary Figure S4**).

### 3.5 Bacterial–fungal co-occurrence networks in salt island soils

**Figure 5:**
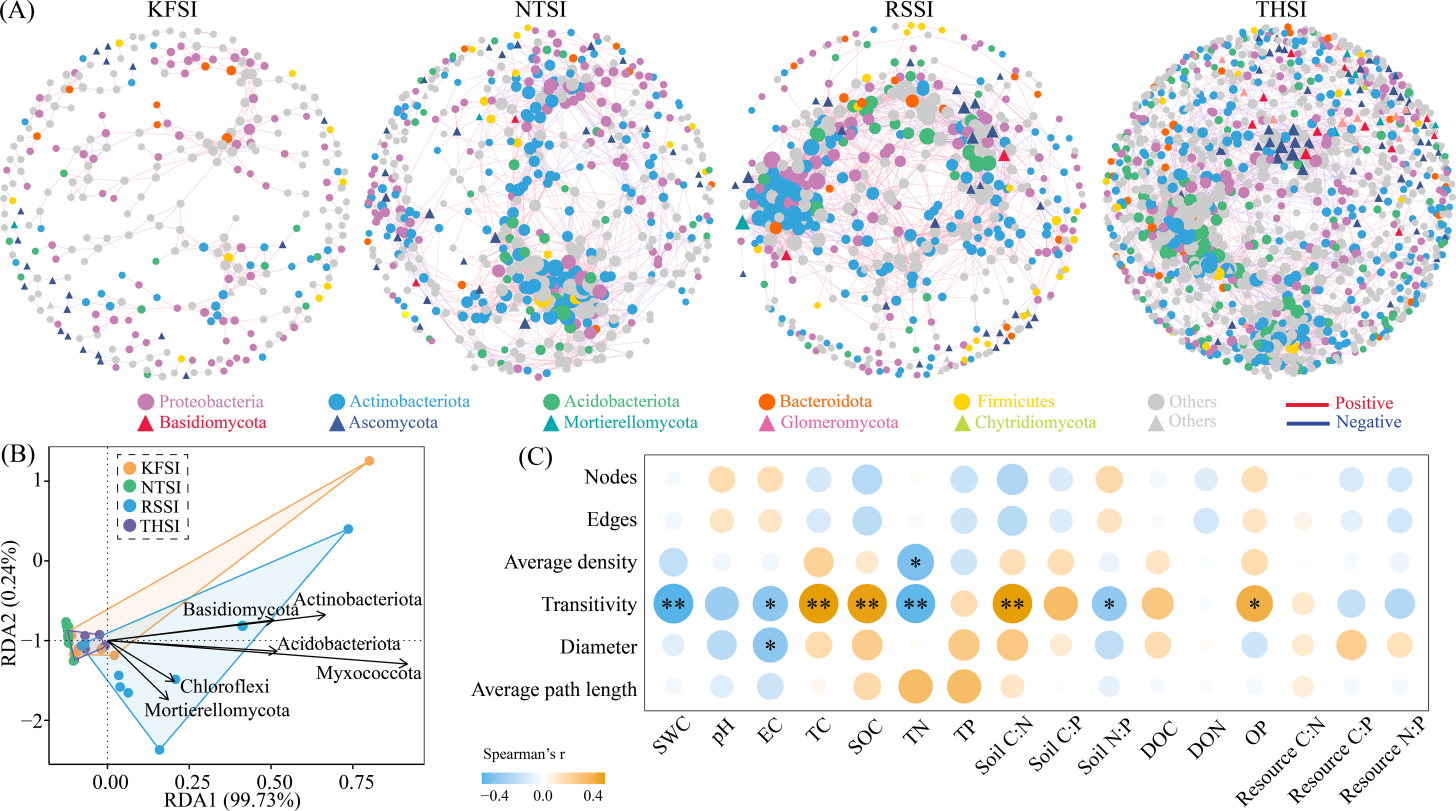
Soil bacterial–fungal co-occurrence network in salt islands **(A)**. RDA of major phyla and network complexity **(B)**. Relationships between network parameters and soil physicochemical properties and ecological stoichiometry **(C)**.

Among the bacterial–fungal co-occurrence networks of the four salt islands in Beichi, the network relationship of KFSI was the simplest, with its index (nodes = 391, edge = 476) being lower than those of NTSI (nodes = 651, edge = 2462), RSSI (nodes = 574, edge = 2779), and THSI (nodes = 1,305, edge = 3929) (**Supplementary Table 2**). Subnetwork analysis revealed that THSI had a higher network complexity, which was significantly higher than that of the other three plots. Actinobacteriota, Acidobacteriota, Myxococcota, and Basidiomycota showed stronger associations with salt island network complexity. Soil SWC, EC, TN, and N:P showed significant negative correlations with network density on the salt islands, while Soil TC, SOC, C:N, and OP showed significant positive correlations (**Figure 5**).

**Figure 6:**
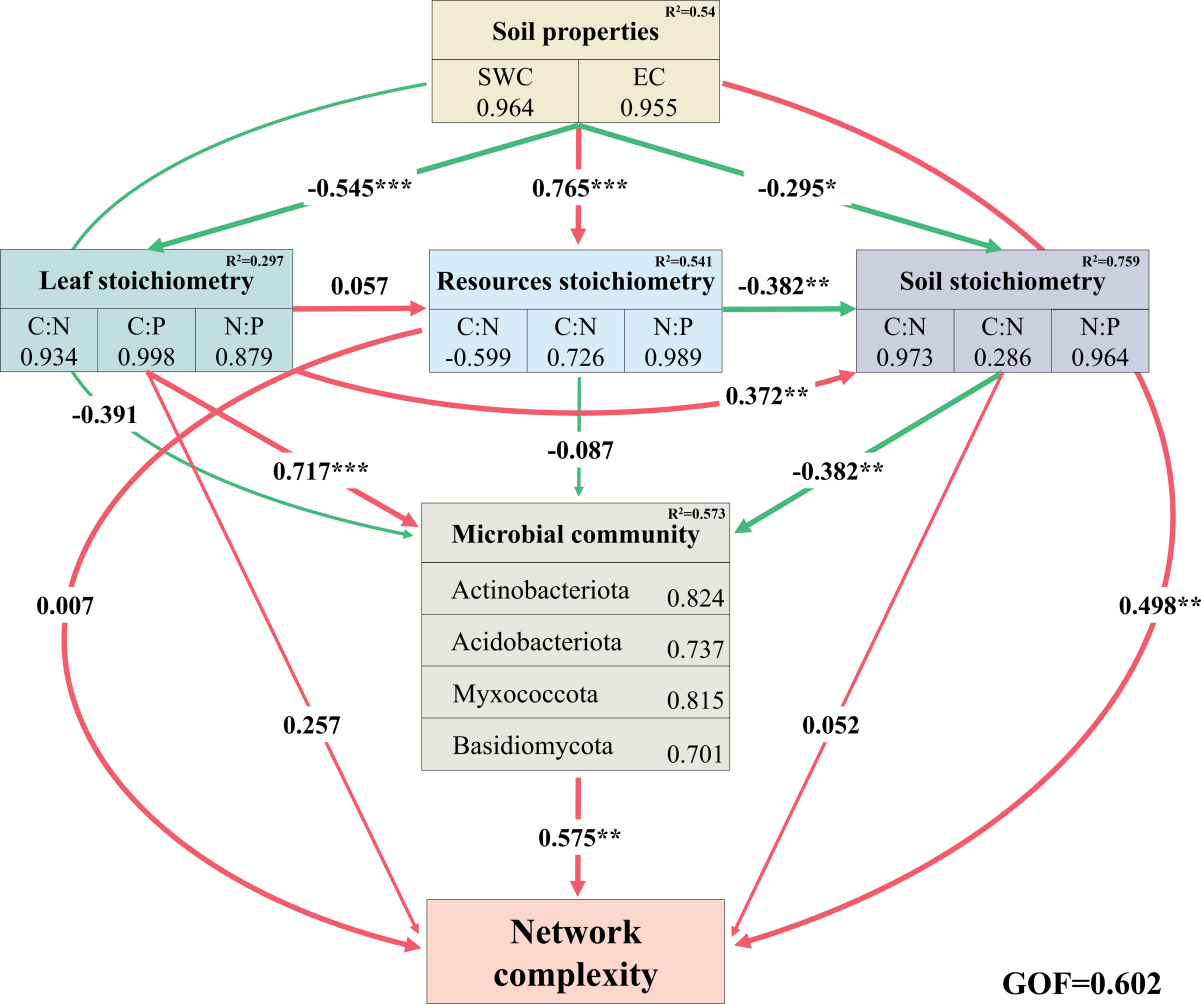
Soil physical properties, leaf stoichiometry, resources stoichiometry, soil stoichiometry, and microbial community pathways for the partial least squares path model analysis of network complexity. Red and green arrows denote positive and negative causal relationships, respectively, with standardized path coefficients displayed above the arrows. The R^2^ values indicate the proportion of variance explained by the model for each variable. (*p < 0.05, **p < 0.01, and ***p < 0.001).

We calculated the NCI based on the network parameters and constructed PLS-PM model. The results showed that soil physical properties (SWC and EC) exhibited significant negative effects on the ecological stoichiometry of halophyte leaves and soil ecological stoichiometry, respectively (*p* < 0.05), while displaying a highly significant positive direct effect on the ecological stoichiometry of soil resources (*p* < 0.001). Concurrently, they also demonstrated a significant positive effect on the complexity of the salt island bacterial–fungal network (*p* < 0.01). The ecological stoichiometry of halophyte leaves exerted direct positive effects on soil ecological stoichiometry and salt island microbial communities (Actinobacteriota, Acidobacteriota, Myxococcota, and Basidiomycota). Furthermore, a significant positive relationship was observed between salt island microbial communities and network complexity (*p* < 0.01) (**Figure 6**).

## 4 Discussion

### 4.1 Changes in water and salinity determine the distribution of halophytes in salt marsh wetlands in arid zones

The distribution of halophytes in arid zones is dependent on water availability. In salt marsh wetlands the water content is adequate in the low marsh wetland but the salinity and ion content are higher. This has been confirmed in many studies, including those on Ebinur Lake in Xinjiang (Niu et al., 2022) and Huamachi Lake in Ningxia (Qiu et al., 2022). Salt-dilution halophytes depend on water, and the absorbed cations are stored in the cellular vesicles of leaves or stems, which is the main characteristic of salt tolerance. This has also been confirmed in studies conducted in Jiantanhu Lake (Pan et al., 2021) and Lianhuachi Lake (Pan et al., 2024a) in Ningxia. As the transect begins in the low marsh (open water) and progresses to the high marsh (near dry land), salt- and drought-tolerant halophytes become the dominant communities because they require a well-developed root system to satisfy their water demands (Eldridge et al., 2011). Similarly, in arid desert ecosystems, wind changes the flow of sand, and shrubs fix the flowing sand owing to their well-developed root systems, gradually forming fertilizer islands (Meglioli et al., 2017). In the present study, in the Beichi salt marsh, the observed plant distribution, extending outward, was *K. foliatum*, *N. tangutorum*, *R. songarica* and *T. hohenackeri*, which is in agreement with the conclusions of previous studies.

As hypothesized, salt islands near salt marsh wetlands had higher salinity, and the water content was also higher in near-wetlands. This water-salt gradient is an important factor in shaping the distribution of plants in salt marsh wetlands in arid zones (Pan et al., 2021). In the present study, the soil pH, EC, Na^+^, Cl^−^, and SO_4_²^-^ of the salt island decreased as the salt marsh wetland extended outward. This has also been confirmed in previous studies, which showed that changes in the moisture and salinity of salt marsh wetlands in arid regions are important factors affecting species distribution (Li et al., 2023). In addition, we observed higher TN and DON contents in the salt island soils of near-salt marsh wetlands, and previous studies have indicated that higher nitrogen accumulation in salt marsh wetlands is the main characteristic of soil nutrients (Wang et al., 2023). Generally, high soil salinity increases TN accumulation by affecting N_2_O emissions (Yang et al., 2020). Most studies have concluded that the wet/dry cycle in salt marsh wetlands caused by seasonal variations increases the accumulation of SOC and TN (Li et al., 2017; Ma et al., 2024). In our study, the *K. foliatum* fertilizer island near the salt marsh had a higher TN content but a lower SOC content than was the case with the other salt islands. We hypothesize that this is influenced by the accumulation of soil salinity because higher salinity reduces the type and amount of plant root exudates (Pan et al., 2022) and inhibits litter decomposition, a phenomenon that is particularly pronounced in arid zones (Jiang et al., 2024).

### 4.2 Stoichiometry of carbon, nitrogen, and phosphorus between halophytes and soil: a coupling relationship

Leaf carbon assimilation is a substrate and energy source for physiological processes during plant growth (Lu et al., 2023a). Further analysis of the leaf chemistry of the four halophytes revealed that TC content was highest in the leaves of *R. songarica* and lowest in *K. foliatum.* We speculate that the lower carbon content of *K. foliatum* leaves may be due to increased energy in the body for cation exchange. According to the energy consumption hypothesis (Shabala et al., 2020), proton pumps that provide the driving force for the transport of cations, such as Na^+^, in halophytes include V-ATPase, V-PPase, and H^+^-PPase (Rea and Sanders, 1987). It also mediates the accumulation of sucrose (which is an important source of carbon) and the transport of inorganic substances (Wang et al., 2013). In addition, when salt saturation occurs in salt-dilution halophytes, chlorophyll content and photosynthetic capacity decrease, thus reducing carbon accumulation (Geissler et al., 2009). The decrease in the chlorophyll content in the leaves of salt-dilution halophytes, which appear red, is due to the accumulation of betacyanin in the leaves, which is why salt marshes appear red in ecological landscapes (Hayakawa and Agarie, 2010). Combined with the results of the present study, it can be confirmed that plant type determined the variation in leaf carbon, nitrogen, and phosphorus contents and their ratios (Lu et al., 2023b). Similarly, we found that the Leaf C, N, and P contents of halophyte leaves were significantly affected by EC, which further indicates that the accumulation of plant leaf nutrients is also affected by changes in the soil environment (Lu et al., 2023a).

Further analysis revealed that *T. hohenackeri* had higher leaf C:N, C:P, and N:P ratios than the other halophytes. In general, phosphorus deficiency limits plant growth and increases leaf C:P and N:P (Zhang et al., 2018). Combined with previous studies, we inferred that the growth of *K. foliatum*, *N. tangutorum*, and *R. songarica* may be more significantly limited by nitrogen (leaf N:P < 14), whereas the growth of *T. hohenackeri* is more significantly limited by phosphorus (leaf N:P > 16) (Koerselman and Meuleman, 1996). Despite the high TP and AP contents of THSI soils in our study, the phosphorus-limiting characteristics of the leaves may be influenced by their leaf salt secretion traits. This is because the salt gland also needs to increase the expression of H^+^-ATPase to drive the transport of Na^+^ from intracellular to extracellular; however, the accumulation of Pi is unfavorable when the soil salt content is low (Chen et al., 2010; Graus et al., 2018). This phenomenon deserves further investigation to determine the relationship between the saline secretion characteristics of halophytes and their Pi requirements.

A strong coupling exists between the carbon and nitrogen ratios in the leaf-soil system of plants (Ren et al., 2024). In the present study, the linear relationship between leaf C:N and soil C:N in the halophyte leaves confirmed these conclusions. We also found a linear relationship between leaf N:P and soil N:P, which may be because of a soil N:P imbalance on leaf N:P. This phenomenon may also increase the nitrogen limitation in plant leaves by changing the soil environment (Zhang et al., 2024). A meta-analysis found that the ratios of carbon to nitrogen and carbon to phosphorus in litter decreased when soil nutrients were enriched (particularly nitrogen) and influenced by water availability (de la Casa et al., 2025). The results of the present study confirm that *K. foliatum* and *N. tangutorum* salt islands have higher SWC and TN, which might be an important reason for the lower leaf C:N ratios found in the leaves of these species. In addition, we found that both leaf C:N and N:P were regulated by SWC in the leaves of different halophytes. Lu et al. (2023a) found that SWC is a key environmental factor affecting the leaf C:N, C:P, and N:P ratios. Thus, plant leaf C:N was positively correlated with soil C:N, while leaf N:P was negatively correlated with soil N:P, indicating that changes in plant leaf nutrients were primarily driven by variations in soil nutrients (Yang Y et al., 2018).

### 4.3 Salt island soil stoichiometry alters soil microbial diversity

The salt island effect based on the “fertilizer island effect”, which is generally observed in perennial halophytic shrubs. In our study of the Beichi salt marsh, we observed a negative correlation between soil salinity and soil nutrients in salt island soils. For example, TC, SOC, TP, and AP in salt island soils showed a significant negative correlation with soil salinity. Previous studies have shown that high soil salinity affects the mineralization and decomposition of organic matter, which in turn has a direct negative effect on soil nutrients (Gao et al., 2022; Lu et al., 2023b). In addition, soil C:N and Resources C:N were also negatively regulated by soil salinity, indicating the sensitivity of soil organic carbon pools to changes in soil salinity (Li et al., 2023). As well, the clear linear relationship between Resources C:N and soil C:N highlights the importance of nutrient concentration (soil dissolved nutrients) in fertilizer islands to changes in soil stoichiometry on salt islands (Luo et al., 2020; Ma et al., 2024). Further analysis of the Beichi soil samples revealed that the Shannon index of bacteria and fungi was higher in THSIs than in other salt islands and that the decrease in soil salinity increased the diversity of soil microorganisms as the salt island of *K. foliatum* extended outward. Moreover, the reduction of soil salinity has led to an obvious “fertilizer island effect” of increased soil nutrients, providing a better habitat for microorganisms to survive (Xu et al., 2021).

The α diversity of bacterial and fungal communities on the KFSI was higher than in areas dominated by other halophytes. This difference may be attributed to the unique ecological functions of *K. foliatum*: its well-developed root system secretes organic acids, which not only alleviate salt stress but also provide diverse carbon sources for microorganisms, thereby supporting a more abundant coexistence of species (Wang et al., 2022). We found that soil TC, SOC, soil C:N, soil C:P and Resources C:N were positively correlated with microbial beta diversity on salt islands through the correlation analysis between physical and chemical properties and microbial diversity on salt islands. This reinforces the importance of soil nutrient changes in salt island soils for microbial community aggregation processes (Xu et al., 2021). It is noteworthy that the Resources N:P of salt island soils was negatively correlated with bacterial and fungal beta diversity. Unlike fertilizer islands, salt islands tend to have higher water, salt, and TN contents near salt marshes, which limits additional nitrogen inputs to the soil, making it easier to deepen the link between soil stoichiometry and microorganisms (Heuck et al., 2015). Furthermore, water variability in desert ecosystems is a constraint on effective phosphorus accumulation in soils, whereas changes in microbial diversity in salt island soils play a pivotal role in the decoupling of carbon, nitrogen, and phosphorus mineralization (Luo et al., 2021; Han et al., 2023).

### 4.4 Salt island ecological stoichiometry governs the complexity of the soil bacterial-fungal co-occurrence network

The complexity and connectivity of bacterial-fungal co-occurrence networks on salt islands also increased with decreasing soil salinity. On the one hand, the networks reflect the strength of microbial interactions to enhance adaptive responses to changes in soil nutrients (Hao et al., 2023); on the other hand, the effect of soil nutrient changes on microbial ecological niche patterns has also been highlighted (Xu et al., 2021). The prevalence of Actinobacteriota, Firmicutes, Acidobacteriota, and network complexity index showed significant linear relationships. In previous studies, the dominant phylum in the network not only maintained the stability of the network but also indirectly affected the stability of the microbial community (Pan et al., 2024a). Notably, the negative impact of soil salinity on microbial network relationships on salt islands can negatively affect geochemical functioning, particularly in salinized soils with unstable bacterial communities (Guan et al., 2021). As the KFSI extends outward, more complex network relationships also help halophytes to better cope with stress and improve the utilization of soil nutrients by the root system (Morriën et al., 2017).

Further analyses revealed that the relative abundances of Acidobacteriota and Myxococcota in the KFSI were significantly higher than those on the other salt islands. This enrichment of Acidobacteriota, a group often associated with oligotrophic conditions (Fierer et al., 2012), may be linked to the observed higher soil C:P ratios in the KFSI (Duan et al., 2024). Acidobacteriota are implicated in soil carbon cycling, with genomic evidence suggesting their capacity to decompose complex organic matter (Xiao et al., 2025). Thus, their increased abundance in the KFSI could play a role in processing recalcitrant carbon pools under these specific stoichiometric conditions. In contrast, Myxococcota can synergistically degrade complex organic matter by secreting extracellular enzymes and forming biofilms to resist osmotic pressure in high-salinity environments (Richy et al., 2024). The relative abundances of Basidiomycota and Mortierellomycota were relatively high on the KFSI and had a high explanatory rate for the physical, chemical, and chemical-metabolic changes in the soil. Basidiomycota have been shown to enhance the nitrogen and phosphorus absorption capacity of *K. foliatum* through exogenous mycorrhizal symbiosis (Tedersoo et al., 2014). It has also been suggested that Mortierellomycota may have potential functions in lipid metabolism and salt ion homeostasis regulation (Wang et al., 2025).

Based on structural equation analysis, differences in soil salinity and nutrients in salt island ecosystems have a direct effect on the leaf stoichiometry of halophytes, which in turn changes the structure of soil microbial communities. In general, soil physical properties (pH and EC) regulate leaf and soil C:N, C:P, and N:P (Song et al., 2024). Soil nutrient status and microbial biogeochemical cycling processes indicate that belowground biological processes depend on the chemical properties of plant leaf litter (Bai et al., 2021). Based on structural equation analysis, differences in soil salinity and nutrients in salt island ecosystems have a direct effect on the leaf stoichiometry of halophytes, which in turn changes the structure of soil microbial communities. In general, soil physical properties (pH and EC) regulate leaf and soil C:N, C:P, and N:P (Song et al., 2024). Soil nutrient status and microbial biogeochemical cycling processes indicate that belowground biological processes depend on the chemical properties of plant leaf litter (Bai et al., 2021). It has also been shown that the combined effects of biotic and abiotic factors on perennial halophytes contribute to shaping salt islands (Ding and Eldridge, 2020; Xiao et al., 2024). We have not only clarified the relationship between ecological stoichiometry, soil microbial communities, and networks of halophyte salt islands but also pointed out the importance of soil C:N for microbial communities and network complexity. As soil physical properties regulate plant–soil stoichiometry, the spatial concentration of soil resources also contributes to the recruitment and shaping of microbial communities (Gao et al., 2022; Song et al., 2024). The positive effect of the microbial community on the network complexity in the structural equation provides positive feedback on the decomposition, mineralization, and redistribution of soil nutrients promoted by salt island microorganisms (Behie and Bidochka, 2014; Cai et al., 2020). Overall, salt islands of halophytic shrubs are active centers of nutrient metabolism and cycling in salt marsh soils in arid zones and play an important ecological role in maintaining the stability of desert ecosystems.

## 5 Conclusion

In this study, we revealed the collaborative evolutionary mechanism of the soil-plant-microbiome system during the formation of salt islands in the Beichi salt marsh. As the transect begins in the low marsh (open water) and progresses to the high marsh (near dry land), soil salinity parameters (SWC, EC, Na^+^, and Cl^-^) decreased from the KFSI to the THSI, whereas SOC accumulated significantly. The TC and SOC contents in the THSI was the highest, which was 1.18 and 2.80 times higher than that of KFSI, respectively. Different halophytes drive leaf stoichiometric differentiation and directly regulate soil ecological stoichiometry through litter input; there is a significant linear correlation between leaf C:N and soil C:N ratios. The microbial community structure is regulated by three factors: soil physicochemical properties (explaining 23.1% of bacterial community variation), plant leaf chemical characteristics (26.7%), and ecological stoichiometric characteristics. The THSI maintained a more complex microbial network (1,305 nodes, 3,929 connections) and high diversity (Shannon index) owing to its high SOC and optimized resource N:P ratio (contributing 26.01%). The PLS-PM confirmed that soil physical properties negatively regulate leaf chemical measurements and positively drive microbial network complexity, forming a cascade effect of “halophytes-soil resources-microbial interactions.” Future research directions should pay closer attention to the role of temporal dynamics—including seasonal shifts in climate and plant phenology—in modulating the soil-plant-microbiome interactions identified here.

## Data Availability

The datasets presented in this study can be found in online repositories. The names of the repository/repositories and accession number(s) can be found in the article/supplementary material.

## Supplementary material

Supplementary Figure 1 Sampling sites in the Beichi salt marsh, Ningxia, China.

Supplementary Table 1 Ecological stoichiometry characteristics of soil C:N:P in salt islands.

Supplementary Figure 2 Shannon and ACE indices of soil bacterial communities **(A)** and fungal communities **(B)** in different salt islands. Also shown are the similarity tests of bacterial **(C)** and fungal **(D)** communities.

Supplementary Figure 3 Correlations between the Shannon, ACE, NMDS1, and NMDS2 indices of bacterial and fungal communities and the leaf characteristics, as well as the soil physicochemical properties.

Supplementary Figure 4 Relative abundance of soil bacterial and fungal phyla based on random forest analysis.

Supplementary Table 2 Soil bacteria-fungi co-occurrence networks indices in salt islands.

Supplementary Figure 5 Network parameters **(A)** and their network complexity index **(B)**.

## References

[B1] Bai Z. Ye J. Wei Y. L. Yan S. K. Yuan H. S. (2021). Soil depth-dependent C/N stoichiometry and fungal and bacterial communities along a temperate forest succession gradient. Catena 207, 105613. doi: 10.1016/j.catena.2021.105613. PMID: 41936479

[B2] Bao S. D. (2000). Soil and agricultural chemistry analysis ( Beijing: China Agriculture Publication).

[B3] Behie S. W. Bidochka M. J. (2014). Nutrient transfer in plant–fungal symbioses. Trends Plant Sci. 19, 734–740. doi: 10.1016/j.tplants.2014.06.007. PMID: 25022353

[B4] Bettinelli M. Beone G. M. Spezia S. Baffi C. (2000). Determination of heavy metals in soils and sediments by microwave-assisted digestion and inductively coupled plasma optical emission spectrometry analysis. Anal. Chim. Acta 424, 289–296. doi: 10.1016/s0003-2670(00)01123-5. PMID: 41334505

[B5] Bokulich N. A. Kaehler B. D. Rideout J. R. Dillon M. Bolyen E. Knight R. . (2018). Optimizing taxonomic classification of marker-gene amplicon sequences with QIIME 2’s q2-feature-classifier plugin. Microbiome 6, 90. doi: 10.1186/s40168-018-0470-z. PMID: 29773078 PMC5956843

[B6] Bolan N. S. Baskaran S. Thiagarajan S. (1996). An evaluation of the methods of measurement of dissolved organic carbon in soils, manures, sludges, and stream water. Commun. Soil Sci. Plan 27, 2723–2737. doi: 10.1080/00103629609369735. PMID: 41909888

[B7] Breshears D. D. Myers O. B. Barnes F. J. (2009). Horizontal heterogeneity in the frequency of plant-available water with woodland intercanopy–canopy vegetation patch type rivals that occuring vertically by soil depth. Ecohydrology 2, 503–519. doi: 10.1002/eco.75. PMID: 41925066

[B8] Cai Y. R. Yan Y. C. Xu D. W. Xu X. L. Wang C. Wang X. . (2020). The fertile island effect collapses under extreme overgrazing: evidence from a shrub-encroached grassland. Plant Soil 448, 201–212. doi: 10.1007/s11104-020-04426-2. PMID: 41933263

[B9] Callahan B. J. McMurdie P. J. Rosen M. J. Han A. W. Johnson A. J. A. Holmes S. P. (2016). DADA2: High-resolution sample inference from Illumina amplicon data. Nat. Methods 13, 581–583. doi: 10.1038/nmeth.3869. PMID: 27214047 PMC4927377

[B10] Caporaso J. G. Lauber C. L. Walters W. A. Berg-Lyons D. Lozupone C. A. Turnbaugh P. J. . (2011). Global patterns of 16S rRNA diversity at a depth of millions of sequences per sample. Proc. Natl. Acad. Sci. 108, 4516–4522. doi: 10.1073/pnas.1000080107 20534432 PMC3063599

[B11] Caporaso J. G. Lauber C. L. Walters W. A. Berg-Lyons D. Huntley J. Fierer N. . (2012). Ultra-high-throughput microbial community analysis on the Illumina HiSeq and MiSeq platforms. ISME J. 6, 1621–1624. doi: 10.1038/ismej.2012.8. PMID: 22402401 PMC3400413

[B12] Chen J. Xiao Q. Wu F. Dong X. He J. Pei Z. . (2010). Nitric oxide enhances salt secretion and Na^+^ sequestration in a mangrove plant, Avicennia marina, through increasing the expression of H^+^-ATPase and Na^+^/H^+^ antiporter under high salinity. Tree Physiol. 30, 1570–1585. doi: 10.1093/treephys/tpq086. PMID: 21030403

[B13] Dassanayake M. Larkin J. C. (2017). Making plants break a sweat: the structure, function, and evolution of plant salt glands. Front. Plant Sci. 8. doi: 10.3389/fpls.2017.00724. PMID: 28400779 PMC5368257

[B14] De Beeck M. O. Lievens B. Busschaert P. Declerck S. Vangronsveld J. Colpaert J. V. (2014). Comparison and validation of some ITS primer pairs useful for fungal metabarcoding studies. PLoS One 9, e97629. doi: 10.1371/journal.pone.0097629 24933453 PMC4059633

[B15] de la Casa J. Sardans J. Galindo M. Peñuelas J. (2025). Stoichiometry of litter decomposition under the effects of climate change and nutrient enrichment: A meta-analysis. Plant Soil 506, 709–726. doi: 10.1007/s11104-024-06718-3. PMID: 41933263

[B16] Delgado-Baquerizo M. Eldridge D. J. Ochoa V. Gozalo B. Singh B. K. Maestre F. T. (2017). Soil microbial communities drive the resistance of ecosystem multifunctionality to global change in drylands across the globe. Ecol. Lett. 20, 1295–1305. doi: 10.1111/ele.12826. PMID: 28921861

[B17] Ding J. Y. Eldridge D. J. (2020). Biotic and abiotic effects on biocrust cover vary with microsite along an extensive aridity gradient. Plant Soil 450, 429–441. doi: 10.1007/s11104-020-04517-0. PMID: 41933263

[B18] Duan X. M. Li J. J. He W. P. Huang J. J. Xiong W. X. Chi S. J. . (2024). Microbial diversity and their extracellular enzyme activities among different soil particle sizes in mossy biocrust under N limitation in the southeastern Tengger Desert, China. Front. Microbiol. 15. doi: 10.3389/fmicb.2024.1328641. PMID: 38357343 PMC10866007

[B19] Eldridge D. J. Bowker M. A. Maestre F. T. Roger E. Reynolds J. F. Whitford W. G. (2011). Impacts of shrub encroachment on ecosystem structure and functioning: towards a global synthesis. Ecol. Lett. 14, 709–722. doi: 10.1111/j.1461-0248.2011.01630.x. PMID: 21592276 PMC3563963

[B20] Elser J. J. Acquisti C. Kumar S. (2011). Stoichiogenomics: the evolutionary ecology of macromolecular elemental composition. Trends Ecol. Evol. 26, 38–44. doi: 10.1016/j.tree.2010.10.006. PMID: 21093095 PMC3010507

[B21] Fierer N. Lauber C. L. Ramirez K. S. Zaneveld J. Bradford M. A. Knight R. (2012). Comparative metagenomic, phylogenetic and physiological analyses of soil microbial communities across nitrogen gradients. ISME J. 6, 1007–1017. doi: 10.1038/ismej.2011.159. PMID: 22134642 PMC3329107

[B22] Flowers T. J. Colmer T. D. (2008). Salinity tolerance in halophytes. New Phytol. 179, 945–963. doi: 10.1086/415032 18565144

[B23] Gao Y. J. Tariq A. Zeng F. J. Sardans J. Peñuelas J. Zhang Z. H. . (2022). Fertile islands” beneath three desert vegetation on soil phosphorus fractions, enzymatic activities, and microbial biomass in the desert-oasis transition zone. Catena 212, 106090. doi: 10.1016/j.catena.2022.106090. PMID: 41936479

[B24] Geissler N. Hussin S. Koyro H. W. (2009). Elevated atmospheric CO_2_ concentration ameliorates effects of NaCl salinity on photosynthesis and leaf structure of Aster tripolium L. J. Exp. Bot. 60, 137–151. doi: 10.1093/jxb/ern271 19036838 PMC3071763

[B25] Graus D. Konrad K. R. Bemm F. Patir Nebioglu M. G. Lorey C. Duscha K. . (2018). High V-PPase activity is beneficial under high salt loads, but detrimental without salinity. New Phytol. 219, 1421–1432. doi: 10.1111/nph.15280. PMID: 29938800 PMC6099232

[B26] Guan Y. P. Jiang N. N. Wu Y. X. Yang Z. Z. Bello A. Yang W. (2021). Disentangling the role of salinity-sodicity in shaping soil microbiome along a natural saline-sodic gradient. Sci. Total Environ. 765, 142738. doi: 10.1016/j.scitotenv.2020.142738. PMID: 33097264

[B27] Haas B. J. Gevers D. Earl A. M. Feldgarden M. Ward D. V. Giannoukos G. . (2011). Chimeric 16S rRNA sequence formation and detection in Sanger and 454-pyrosequenced PCR amplicons. Genome Res. 21, 494–504. doi: 10.1101/gr.112730.110. PMID: 21212162 PMC3044863

[B28] Hair J. F. Sarstedt M. Ringle C. M. Mena J. A. (2012). An assessment of the use of partial least squares structural equation modeling in marketing research. J. Acad. Market 40, 414–433. doi: 10.1007/s11747-011-0261-6. PMID: 41933263

[B29] Han X. M. Zhai K. Y. Liu S. G. Chen H. Y. He Y. H. Du Z. G. . (2023). Forest restoration decouple soil C:N:P stoichiometry but has little effects on microbial biodiversity globally. J. Sustain. Agric. Environ. 2, 468–478. doi: 10.1002/sae2.12084. PMID: 41925066

[B30] Hao J. Q. Xu W. Song J. J. Gao G. X. Bai J. Z. Yu Q. . (2023). Adaptability of agricultural soil microbial nutrient utilization regulates community assembly under mulching measures on the Loess Plateau. Agr Ecosyst. Environ. 357, 108702. doi: 10.1016/j.agee.2023.108702. PMID: 41936479

[B31] Hayakawa K. Agarie S. (2010). Physiological roles of betacyanin in a halophyte, suaeda japonica makino. Plant Prod. Sci. 13, 351–359. doi: 10.1626/pps.13.351

[B32] He H. Bleby T. M. Veneklaas E. J. Lambers H. (2011). Dinitrogen-fixing Acacia species from phosphorus-impoverished soils resorb leaf phosphorus efficiently. Plant Cell Environ. 34, 2060–2070. doi: 10.1111/j.1365-3040.2011.02403.x. PMID: 21819412

[B33] Heuck C. Weig A. Spohn M. (2015). Soil microbial biomass C:N:P stoichiometry and microbial use of organic phosphorus. Soil Biol. Biochem. 85, 119–129. doi: 10.1016/j.soilbio.2015.02.029. PMID: 41936479

[B34] Jiang X. W. Chen F. Yang J. J. Zhou Z. L. Han L. Lyu R. H. (2024). Decomposition of foliar litter from dominant plants of desert riparian forests in extremely arid regions. Forests 15, 949. doi: 10.3390/f15060949. PMID: 41725453

[B35] Jiao S. Chen W. M. Wang J. L. Du N. N. Li Q. P. Wei G. H. (2018). Soil microbiomes with distinct assemblies through vertical soil profiles drive the cycling of multiple nutrients in reforested ecosystems. Microbiome 6, 146. doi: 10.1186/s40168-018-0526-0. PMID: 30131068 PMC6104017

[B36] Kang P. Pan Y. Q. Hu J. P. Qu X. Ji Q. B. Zhuang C. Y. . (2025). Straw mulch and orchard grass mediate soil microbial nutrient acquisition and microbial community composition in *Ziziphus jujuba* orchard. Plant Soil 512, 1203–1219. doi: 10.1007/s11104-024-07144-1

[B37] Kõljalg U. Larsson K. H. Abarenkov K. Nilsson R. H. Alexander I. J. Eberhardt U. . (2005). UNITE: a database providing web-based methods for the molecular identification of ectomycorrhizal fungi. New Phytol 166, 1063–1068. doi: 10.1111/j.1469-8137.2005.01376.x 15869663

[B38] Knapp A. K. Briggs J. M. Collins S. L. Archer S. R. Bret-Harte M. S. Ewers B. E. . (2008). Shrub encroachment in North American grasslands: shifts in growth form dominance rapidly alters control of ecosystem carbon inputs. Global Change Biol. 14, 615–623. doi: 10.1111/j.1365-2486.2007.01512.x. PMID: 41940437

[B39] Koerselman W. Arthur F. M. M. (1996). The vegetation N:P ratio: a new tool to detect the nature of nutrient limitation. J. Appl. Ecol. 33, 1441–1450. doi: 10.2307/2404783

[B40] Li S. Y. Chen W. M. Li Z. B. Bu L. Y. Jin Z. X. Wei G. H. . (2021). Fertile islands lead to more conspicuous spatial heterogeneity of bacteria than soil physicochemical properties in a desert ecosystem. Catena 206, 105526. doi: 10.1016/j.catena.2021.105526. PMID: 41936479

[B41] Li X. Y. Wen B. L. Yang F. Hartley A. Li X. J. (2017). Effects of alternate flooding–drought conditions on degenerated Phragmites australis salt marsh in Northeast China. Restor. Ecol. 25, 810–819. doi: 10.1111/rec.12500

[B42] Li S. Y. Yang S. S. Wei X. M. Jiao S. Luo W. Chen W. M. . (2023). Reduced trace gas oxidizers as a response to organic carbon availability linked to oligotrophs in desert fertile islands. ISME J. 17, 1257–1266. doi: 10.1038/s41396-023-01437-6. PMID: 37253970 PMC10356767

[B43] Liu Z. K. Shao Y. Y. Cui Q. G. Ye X. H. Huang Z. Y. (2024). ‘Fertile island’ effects on the soil microbial community beneath the canopy of Tetraena mongolica, an endangered and dominant shrub in the West Ordos Desert, North China. BMC Plant Biol. 24, 178. doi: 10.1186/s12870-024-04873-4 38454326 PMC10921620

[B44] Lu J. N. Feng S. Wang S. K. Zhang B. L. Ning Z. Y. Wang R. X. . (2023a). Patterns and driving mechanism of soil organic carbon, nitrogen, and phosphorus stoichiometry across northern China’s desert-grassland transition zone. Catena 220, 106695. doi: 10.1016/j.catena.2022.106695. PMID: 41936479

[B45] Lu J. N. Zhao X. Y. Wang S. K. Feng S. Ning Z. Y. Wang R. X. . (2023b). Untangling the influence of abiotic and biotic factors on leaf C, N, and P stoichiometry along a desert-grassland transition zone in northern China. Sci. Total Environ. 884, 163902. doi: 10.1016/j.scitotenv.2023.163902. PMID: 37137371

[B46] Luo X. Z. Hou E. Q. Chen J. Q. Li J. Zhang L. L. Zang X. W. . (2020). Dynamics of carbon, nitrogen, and phosphorus stocks and stoichiometry resulting from conversion of primary broadleaf forest to plantation and secondary forest in subtropical China. Catena 193, 104606. doi: 10.1016/j.catena.2020.104606. PMID: 41936479

[B47] Luo Y. Peng Q. W. Li K. H. Gong Y. M. Liu Y. Y. Han W. X. (2021). Patterns of nitrogen and phosphorus stoichiometry among leaf, stem and root of desert plants and responses to climate and soil factors in Xinjiang, China. Catena 199, 105100. doi: 10.1016/j.catena.2020.105100. PMID: 41936479

[B48] Ma B. Wang H. Z. Dsouza M. Lou J. He Y. Dai Z. M. . (2016). Geographic patterns of co-occurrence network topological features for soil microbiota at continental scale in eastern China. ISME J. 10, 1891–1901. doi: 10.1038/ismej.2015.261. PMID: 26771927 PMC5029158

[B49] Ma Z. W. Wu Y. N. Zhao S. Q. Pan Y. Y. Liu J. K. Zhang M. X. . (2024). The role of tidal creeks in shaping carbon and nitrogen patterns in a Chinese salt marsh. Front. Mar. Sci. 11. doi: 10.3389/fmars.2024.1361474. PMID: 41930257

[B50] Magoč T. Salzberg S. L. (2011). FLASH: fast length adjustment of short reads to improve genome assemblies. Bioinformatics 27, 2957–2963. doi: 10.1093/bioinformatics/btr507 21903629 PMC3198573

[B51] Meglioli P. A. Aranibar J. N. Villagra P. E. Riveros C. V. (2017). Spatial patterns of soil resources under different land use in Prosopis woodlands of the Monte desert. Catena 149, 86–97. doi: 10.1016/j.catena.2016.09.002. PMID: 41936479

[B52] Mooshammer M. Wanek W. Schnecker J. Wild B. Leitner S. Hofhansl F. . (2012). Stoichiometric controls of nitrogen and phosphorus cycling in decomposing beech leaf litter. Ecology 93, 770–782. doi: 10.1890/11-0721.1. PMID: 22690628

[B53] Morriën E. Hannula S. E. Snoek L. B. Helmsing N. R. Zweers H. de Hollander M. . (2017). Soil networks become more connected and take up more carbon as nature restoration progresses. Nat. Commun. 8, 14349. doi: 10.1038/ncomms14349 28176768 PMC5309817

[B54] Niu Y. H. Hu W. G. Zhou T. T. He B. Chen X. M. Li Y. (2022). Diversity of nirS and nirK denitrifying bacteria in rhizosphere and non-rhizosphere soils of halophytes in Ebinur Lake Wetland. Bio/Technol. Biotec Eq 36, 209–219. doi: 10.62517/jlsa.202507410

[B55] Olsen S. R. (1954). “ Estimation of available phosphorus in soils by extraction with sodium bicarbonate,” in United states department of agriculture ( United States Department of Agriculture, Washington).

[B56] Pan Y. Q. Kang P. Hu J. P. Song N. P. (2021). Bacterial community demonstrates stronger network connectivity than fungal community in desert-grassland salt marsh. Sci. Total Environ. 798, 149118. doi: 10.1016/j.scitotenv.2021.149118. PMID: 34332392

[B57] Pan Y. Q. Kang P. Qu X. Zhang H. X. Li X. R. (2024a). Response of the soil bacterial community to seasonal variations and land reclamation in a desert grassland. Ecol. Indic. 165, 112227. doi: 10.1016/j.ecolind.2024.112227. PMID: 41936479

[B58] Pan Y. Q. Kang P. Tan M. Hu J. P. Zhang Y. Q. Zhang J. L. . (2022). Root exudates and rhizosphere soil bacterial relationships of Nitraria tangutorum are linked to k-strategists bacterial community under salt stress. Front. Plant Sci. 13-2022. doi: 10.3389/fpls.2022.997292. PMID: 36119572 PMC9471988

[B59] Pan Y. Q. Kang P. Zhang Y. Q. Li X. R. (2024b). *Kalidium cuspidatum* colonization changes the structure and function of salt crust microbial communities. Environ. Sci. pollut. R. 31, 19764–19778. doi: 10.1007/s11356-024-32364-4 38363505

[B60] Qiu L. P. Kong W. B. Zhu H. S. Zhang Q. Banerjee S. Ishii S. . (2022). Halophytes increase rhizosphere microbial diversity, network complexity and function in inland saline ecosystem. Sci. Total Environ. 831, 154944. doi: 10.1016/j.scitotenv.2022.154944. PMID: 35367547

[B61] Qu X. Pan Y. Q. Wang P. Q. Ran L. L. Qin G. F. Li Q. F. . (2024). Response of phyllosphere and rhizosphere microbial communities to salt stress of Tamarix chinensis. Plants 13, 1091. doi: 10.3390/plants13081091. PMID: 38674498 PMC11054833

[B62] Racine J. S. (2012). RStudio: a platform-independent IDE for R and sweave. J. Appl. Economet 27, 167–172. doi: 10.1002/jae.1278. PMID: 41925066

[B63] Rea P. A. Sanders D. (1987). Tonoplast energization: Two H^+^ pumps, one membrane. Physiol. Plant 71, 131–141. doi: 10.1111/j.1399-3054.1987.tb04630.x. PMID: 41940437

[B64] Ren Y. Gao G. L. Ding G. D. Zhang Y. Zhao P. S. (2024). Patterns and environmental drivers of C, N, and P stoichiometry in the leaf-litter-soil system associated with Mongolian pine forests. Ecol. Evol. 14, e11172. doi: 10.1002/ece3.11172. PMID: 38516573 PMC10954427

[B65] Richy E. Thiago Dobbler P. Tláskal V. López-Mondéjar R. Baldrian P. Kyselková M. (2024). Long-read sequencing sheds light on key bacteria contributing to deadwood decomposition processes. Environ. Microbiome 19, 99. doi: 10.1186/s40793-024-00639-5. PMID: 39627869 PMC11613949

[B66] Ridolfi L. Laio F. D’Odorico P. (2008). Fertility island formation and evolution in dryland ecosystems. Ecol. Soc 13, 130105. doi: 10.5751/es-02302-130105. PMID: 30174746

[B67] Shabala S. Chen G. Chen Z. H. Pottosin I. (2020). The energy cost of the tonoplast futile sodium leak. New Phytol 225, 1105–1110. doi: 10.1111/nph.15758 30802968

[B68] Shannon P. Markiel A. Ozier O. Baliga N. S. Wang J. T. Ramage D. . (2003). Cytoscape: a software environment for integrated models of biomolecular interaction networks. Genome Res. 13, 2498–2504. doi: 10.1101/gr.1239303. PMID: 14597658 PMC403769

[B69] Song Z. B. Zuo X. A. Zhao X. Y. Qiao J. J. Ya H. Li X. Y. . (2024). Plant functional traits mediate the response magnitude of plant-litter-soil microbial C: N: P stoichiometry to nitrogen addition in a desert steppe. Sci. Total Environ. 915, 169915. doi: 10.1016/j.scitotenv.2024.169915. PMID: 38190901

[B70] Sunagawa S. Coelho L. P. Chaffron S. Kultima J. R. Labadie K. Salazar G. . (2015). Structure and function of the global ocean microbiome. Science 348, 1261359. doi: 10.1126/science.1261359. PMID: 25999513

[B71] Tedersoo L. Bahram M. Põlme S. Kõljalg U. Yorou N. S. Wijesundera R. . (2014). Global diversity and geography of soil fungi. Science 346, 1256688. doi: 10.1126/science.1256688. PMID: 25430773

[B72] Wang X. C. Chang L. L. Wang B. C. Wang D. Li P. H. Wang L. M. . (2013). Comparative proteomics of Thellungiella halophila leaves from plants subjected to salinity reveals the importance of chloroplastic starch and soluble sugars in halophyte salt tolerance*. Mol. Cell. Proteomics 12, 2174–2195. doi: 10.1016/j.jprot.2013.03.024. PMID: 23660471 PMC3734578

[B73] Wang R. M. Cui L. J. Li J. Li W. (2023). Factors driving the halophyte rhizosphere bacterial communities in coastal salt marshes. Front. Microbiol. 14 - 2023. doi: 10.3389/fmicb.2023.1127958. PMID: 36910212 PMC9992437

[B74] Wang B. C. Kuang S. P. Shao H. B. Cheng F. Wang H. H. (2022). Improving soil fertility by driving microbial community changes in saline soils of Yellow River Delta under petroleum pollution. J. Environ. Manage. 304, 114265. doi: 10.1016/j.jenvman.2021.114265. PMID: 34915391

[B75] Wang Y. Y. Lu Q. Y. Zhang F. Wang W. Wu C. Y. (2025). Effects of biochar on the yield of melon and the diversity of rhizosphere soil microbial communities under saline–alkali stress. Plants 14, 1423. doi: 10.3390/plants14101423. PMID: 40430987 PMC12115172

[B76] Xiao Y. X. Song B. Y. Galip N. Zhang X. Y. Zhuang W. W. (2024). The ‘fertilizer island effect’ cannot eliminate competition between leguminous shrubs and symbiotic herbs in desert ecosystems. Catena 246, 108429. doi: 10.1016/j.catena.2024.108429. PMID: 41936479

[B77] Xiao R. Zhang J. H. Cheng Y. H. Li T. F. Zhao C. Y. Zhang L. Y. (2025). Impact of phosphorus reduction combined with biofertilizer application on soil nutrients and microbial communities in arid oasis agricultural areas. Front. Microbiol. 16. doi: 10.3389/fmicb.2025.1606813. PMID: 41113633 PMC12533544

[B78] Xu H. W. Wang X. K. Qu Q. Yang Z. Y. Wang M. G. Liu G. B. . (2021). Variations and factors characterizing ecological niches of species in a stable grassland plant community. Ecol. Indic. 128, 107846. doi: 10.1016/j.ecolind.2021.107846. PMID: 41936479

[B79] Yang B. Li X. Z. Lin S. W. Xie Z. L. Yuan Y. Q. Espenberg M. . (2020). Invasive Spartina alterniflora can mitigate N_2_O emission in coastal salt marshes. Ecol. Eng. 147, 105758. doi: 10.53347/rid-6722

[B80] Yang Y. Liu B. R. An S. S. (2018). Ecological stoichiometry in leaves, roots, litters and soil among different plant communities in a desertified region of Northern China. Catena 166, 328–338. doi: 10.1016/j.catena.2018.04.018. PMID: 41936479

[B81] Yang B. M. Wang R. S. Xiao H. J. Cao Q. Q. Liu T. (2018). Spatio-temporal variations of soil water content and salinity around individual Tamarix ramosissima in a semi-arid saline region of the upper Yellow River, Northwest China. J. Arid. Land 10, 101–114. doi: 10.1007/s40333-017-0072-9. PMID: 41933263

[B82] Yilmaz P. Parfrey L. W. Yarza P. Gerken J. Pruesse E. Quast C. . (2014). The SILVA and “All-species Living Tree Project (LTP)” taxonomic frameworks. Nucleic Acids Res 42, 643–648. doi: 10.1093/nar/gkt1209 24293649 PMC3965112

[B83] Yuan F. Leng B. Wang B. (2016). Progress in studying salt secretion from the salt glands in recretohalophytes: how do plants secrete salt? Front. Plant Sci. 7. doi: 10.3389/fpls.2016.00977. PMID: 27446195 PMC4927796

[B84] Zhang H. Li X. Wang S. Q. Jiang C. Y. Cui Y. H. Fan R. Y. . (2024). Tree–litter–soil system C:N:P stoichiometry and tree organ homeostasis in mixed and pure Chinese fir stands in south subtropical China. Front. Forests Glob. Change 7 - 2024. doi: 10.3389/ffgc.2024.1293439. PMID: 41930257

[B85] Zhang Y. B. Wang H. F. Cai Y. Yang Q. Lv G. H. (2022). Fertile island effect by three typical woody plants on wetlands of Ebinur Lake, northwestern China. Front. Environ. Sci. 10 - 2022. doi: 10.3389/fenvs.2022.1034077. PMID: 41930257

[B86] Zhang H. Wang J. N. Wang J. Y. Guo Z. W. Wang G. G. Zeng D. H. . (2018). Tree stoichiometry and nutrient resorption along a chronosequence of Metasequoia glyptostroboides forests in coastal China. For. Ecol. Manag 430, 445–450. doi: 10.1016/j.foreco.2018.08.037. PMID: 41936479

[B87] Zhao K. F. Song J. Feng G. Zhao M. Liu J. P. (2011). Species, types, distribution, and economic potential of halophytes in China. Plant Soil 342, 495–509. doi: 10.1007/s11104-010-0470-7. PMID: 41933263

