# Supplementary material for: Changes in soil ecological stoichiometry and microbial communities related to leaf stoichiometry of different halophytes
Source: Front Plant Sci. 2026 May 8;17:1793742. doi: 10.3389/fpls.2026.1793742 (PMC13196673; doi:10.3389/fpls.2026.1793742)
Supplement: Supplementary Figure 1 — Sampling sites in the Beichi salt marsh, Ningxia, China. [file Table1.docx]

**Table S1** Ecological stoichiometry characteristics of soil C:N:P in salt islands.

|  | KFSI | NTSI | RSSI | THSI |
| --- | --- | --- | --- | --- |
| Soil C:N | 1.53±0.22**c** | 4.51±0.55**b** | 5.27±0.74**b** | 8.62±0.66**a** |
| Soil C:P | 4.33±0.55**b** | 11.11±1.88**a** | 9.33±2.15**a** | 10.41±0.77**a** |
| Soil N:P | 3.03±0.32**a** | 2.53±0.38**a** | 1.62±0.17**b** | 1.22±0.06**b** |
| Resources C:N | 49.04±3.71**b** | 87.44±11.31**a** | 81.02±4.67**a** | 85.94±7.53**a** |
| Resources C:P | 87.98±9.54**a** | 74.87±12.58**a** | 62.23±9.44**a** | 59.71±5.00**a** |
| Resources N:P | 1.82±0.16**a** | 0.91±0.15**b** | 0.85±0.14**b** | 0.72±0.07**b** |

**Table S2** Soil bacteria-fungi co-occurrence networks indices in salt islands.

|  | KFSI | NTSI | RSSI | THSI |
| --- | --- | --- | --- | --- |
| Number nodes | 391 | 651 | 574 | 1305 |
| Number edges | 476 | 2462 | 2779 | 3929 |
| Modularity | 0.8479 | 0.5866 | 0.6061 | 0.7004 |
| Average path length | 7.8014 | 4.6142 | 4.068 | 6.2669 |
| Graph diameter | 20.3984 | 12.8981 | 15.5922 | 18.42389 |
| Graph density | 0.0062 | 0.0116 | 0.0168 | 0.00461 |
| Clustering coefficient | 0.4706 | 0.4516 | 0.4684 | 0.3987 |
| Betweenness centralization | 0.0968 | 0.0325 | 0.0370 | 0.0265 |
| Degree centralization | 0.0296 | 0.0529 | 0.0668 | 0.0261 |
